# Molecular insights into Zeaxanthin-dependent quenching in higher plants

**DOI:** 10.1038/srep13679

**Published:** 2015-09-01

**Authors:** Pengqi Xu, Lijin Tian, Miroslav Kloz, Roberta Croce

**Affiliations:** 1 Biophysics of Photosynthesis, Department of Physics and Astronomy, Faculty of Sciences, VU University Amsterdam and LaserLab Amsterdam. De Boelelaan, 1081, 1081 HV, Amsterdam, The Netherlands

## Abstract

Photosynthetic organisms protect themselves from high-light stress by dissipating excess absorbed energy as heat in a process called non-photochemical quenching (NPQ). Zeaxanthin is essential for the full development of NPQ, but its role remains debated. The main discussion revolves around two points: where does zeaxanthin bind and does it quench? To answer these questions we have followed the zeaxanthin-dependent quenching from leaves to individual complexes, including supercomplexes. We show that small amounts of zeaxanthin are associated with the complexes, but in contrast to what is generally believed, zeaxanthin binding *per se* does not cause conformational changes in the complexes and does not induce quenching, not even at low pH. We show that in NPQ conditions zeaxanthin does not exchange for violaxanthin in the internal binding sites of the antennas but is located at the periphery of the complexes. These results together with the observation that the zeaxanthin-dependent quenching is active in isolated membranes, but not in functional supercomplexes, suggests that zeaxanthin is acting in between the complexes, helping to create/participating in a variety of quenching sites. This can explain why none of the antennas appears to be essential for NPQ and the multiple quenching mechanisms that have been observed in plants.

The capture and storage of light energy by photosynthetic organisms is the process that sustains virtually all life on earth, but it is also a risky business. The simultaneous presence of excitation energy and molecular oxygen, as it occurs in the membranes of plants, may lead to the formation of reactive oxygen species resulting in photodamage. To avoid photodamage, photosynthetic organisms have evolved a series of protective mechanisms^1^, the fastest of which is the regulation of the light-harvesting capacity of the photosystems (PS) via the dissipation of the excess absorbed energy as heat in a process called non-photochemical quenching (NPQ)^2^.

PSI and PSII are multi-protein membrane complexes that coordinate chlorophyll (Chl) and carotenoid molecules. Both complexes are composed of (i) a core, responsible for charge separation and electron transport, and (ii) an outer antenna, which increases the absorption cross section of the core^3^. In higher plant, the outer antenna is composed of members of the light-harvesting complex (Lhc) multigenic family, which together with the cores form supercomplexes. In the PSII supercomplex, the core (C) is surrounded by one copy each of the minor antennas CP29, CP26 and CP24 and by several LHCII trimers^4^. The largest PSII supercomplex purified from *A. thaliana* contains two LHCII trimers per core, which are indicated as S (Strongly bound) and M (Moderately bound) forming the C_2_S_2_M_2_ complex that has a molecular weight of 1400 KDa and coordinates around 300 Chls^4^. In the PSI supercomplex, (600 KDa and 170 Chls), 4 Lhca (Lhca1-4) are located on one side of the core^5^.

Lhcs have a conserved structure with three transmembrane helices and coordinate a maximum of 14 Chls per monomer: 5–9 Chl *a* and 4–6 Chl *b*^6^,^7^,^8^. Two carotenoid binding sites, named L1 and L2, are present in all Lhcs and in normal conditions coordinate lutein (Lut) and violaxanthin (Vio). Two additional sites, N1 and V1, located at the periphery of the complexes, are present only in some Lhcs and coordinate neoxanthin (Neo) and Vio/Lut, respectively^6^,^9^. The xanthophylls in the V1 site are loosely bound and they do not participate in excitation energy transfer and triplet quenching^9^,^10^.

It is generally believed that the Lhcs have the ability to switch from a light-harvesting to a quenched conformation, to perform different functions in different conditions, i.e. collecting photons and transferring excitation energy to the core in limiting light conditions and protecting from photodamage by participating to the NPQ processes in high light^2^.

NPQ is triggered by the acidification of the thylakoid lumen, that in plants induces two events: (1) activation of PsbS, a protein essential for NPQ^11^, which occurs in seconds; and (2) activation of violaxanthin de-epoxidase, which leads to the production of zeaxanthin (Zea), and occurs within minutes^12^,^13^.

The current view is that Zea substitutes Vio in the V1 site of LHCII and in the L2 site of the minor antennas, inducing a conformational change that stabilizes the quenched state of the complexes^14^,^15^,^16^. Different hypotheses have been put forward regarding the role of zeaxanthin in the quenching: (i) it can act as an allosteric effector^17^, with the binding of Zea to LHCII leading to a change in the pK values of protonable residues, lowering the threshold for quenching activation^2^; (ii). It acts as a quencher^18^ via the formation of a radical cation on the L2 site of the minor antennas^19^,^20^, or via a Chl-carotenoid interaction in LHCII^21^ mediated by PsbS^22^. Using recombinant proteins and complexes purified from the *npq*2 mutant of *A. thaliana*, in which Zea is constitutively present^13^, it was shown that Zea is able to bind to the L1 and L2 sites of the Lhcs^14^,^23^,^24^ and that this binding induces quenching in isolated Lhcs^19^,^25^,^26^. But, is this what happens in light stress (NPQ) conditions? When complexes were purified from stressed plants only a small amount of Zea was found associated with them^9^,^14^,^21^,^27^,^28^,^29^. Around 0.15 Zea molecules were present in the V1 site of LHCII^30^, but contrasting results were obtained regarding its effect on quenching^9^,^21^,^31^. Small quantities of Zea were also found associated with the minor antennas. However, these results were difficult to interpret because the harsh methods used in the purification leaded to pigments loss^9^,^30^. Moreover, during purification, part of the Zea might be re-converted to Vio^30^, making it difficult to evaluate the real extent of its binding to the complexes and its functional role.

In this work, we have envisaged ways to overcome those problems and we have monitored the zeaxanthin-quenching activity going from leaves to isolated complexes via membranes and functional photosystems.

## Results

**Figure 1:**
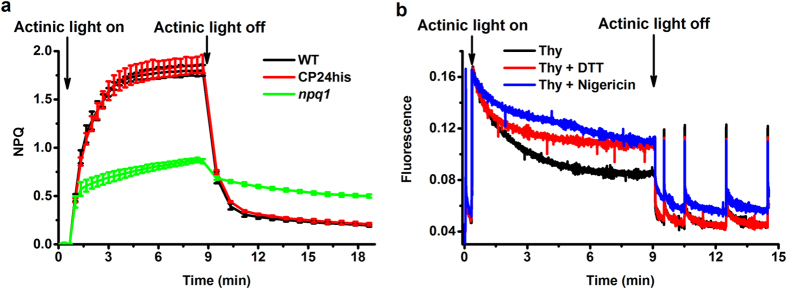
NPQ measurement of leave and Fluorescence yield measurement of isolated thylakoid membranes. (**a**) NPQ measurements in leaves. (**b**) NPQ induction in isolated thylakoids. Chlorophyll fluorescence was performed with a Dual PAM 100 chlorophyll fluorescence photosynthesis analyzer (Heinz Walz). Before measurements, the plants were dark-adapted for at least 45 min. The measuring light was 3 μmol photons m^−2^ s^−1^, and the saturating light pulses 5000 μmol photons m^−2^ s^−1^ (duration 500 ms). Actinic light of 1000 μmol photons m^−2^ s^−1^ was used.

Figure 1 shows that zeaxanthin-dependent quenching is active at the level of leaves (Fig. 1a) and of purified thylakoids (Fig. 1b). To determine where the active Zea is located we then proceed with the isolation of the components of the photosynthetic apparatus. The complexes were isolated from plants treated with 30 min of high light (stress, NPQ conditions), which induces the maximum of Zea synthesis^32^ and from plants grown in the same conditions without high light treatment (control), which do not contain Zea (see M&M for details). In these conditions NPQ is stable at the maximum level e.g.^33^. To avoid the activation of the Zea epoxidase during purification, all steps involving light stressed plants were performed at 4 °C and pH 5.5. The almost identical de-epoxidation level (DEPS) of leaves and thylakoid membrane (0.62 and 0.60 respectively) indicates that this strategy was successful in inhibiting the reconversion of Zea to Vio.

**Figure 2:**
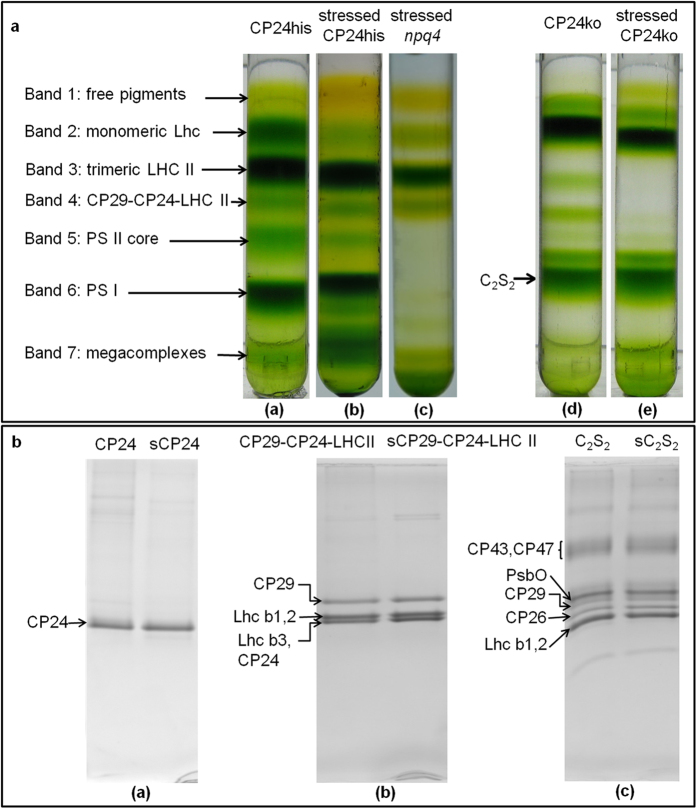
Purification of the photosynthetic complexes (a) Sucrose gradients. The name of the plant line from which the membranes were prepared is reported on top of each tube. (**a**) and (**b**) contain thylakoid membranes solubilized with 0.6% α-DDM; (**c**), (**d**) and (**e**) contain grana membranes solubilized with 0.3% α-DDM. To optimize the purification of the individual complexes sucrose gradients with different density were used: 0–1 M in (**a–c**) and 0–1.3 M in (**d**) and (**e**), which explains the different mobility of the complexes in the gradients (e.g. the PSII supercomplexes are in the pellet in (**c**)). (**b**) SDS-PAGE showing the protein composition of the relevant purified complexes from control and stressed (s) plants. (**a**). CP24 (tagged); (**b**). CP29-CP24 (tagged)-LHCII complex; (**c**). PS II supercomplex.

The pigment-protein complexes were purified by mild solubilization of thylakoid and grana membranes followed by sucrose gradients (Fig. 2a). Small differences in the band patterns from control and light stressed plants were mainly due to the presence of a higher amount of megacomplexes upon stress, which results in a higher intensity of the bands of Lhc monomers, trimers and PSII core in the control gradients. Band 4 was observed only in a few cases after light stress, indicating that in those conditions it is very unstable as previously reported^34^. The free pigment band, which is enriched in carotenoids, was more intense in stressed membranes, in agreement with a lower Chl/car ratio of these membranes and was enriched in Zea and Lut (Supplementary Table S2). On the contrary little Vio was present in this band suggesting that most of the “free” Vio was de-epoxidated to Zea.

Homogeneous preparations of LHCII trimers were obtained directly from the thylakoid gradients while the bands containing all other complexes were not homogeneous enough for our purpose and needed to be further purified. Note that to investigate to which complexes and in which sites Zea binds, and the effect of this binding on the properties of the complexes, it was essential to obtain pure and homogeneous preparations, but also to use mild procedures to avoid pigment loss. We thus developed different strategies to fulfill these requirements.

CP24 and the CP29-CP24-LHCII complex were purified, loading the corresponding bands of the sucrose gradient on a Ni-affinity column, taking advantage of a His tag on CP24^8^. In addition the CP29-CP24-LHCII complex was also purified from the grana membranes of the *npq4* mutant, that maintains the connection between these antennas also in stress conditions^34^. The two preparations had identical properties.

The PSII supercomplexes C_2_S_2_, which is composed of a dimeric core and two copies each of CP29, CP26 and LHCII trimer S, were obtained by very mild solubilization of the grana membranes^4^. To avoid the presence of supercomplexes with different protein composition, which is a direct consequence of the PSII organization in WT plants^4^, C_2_S_2_ was purified from the CP24ko mutant, which does not contain LHCII trimer M and can thus not form C_2_S_2_M_2_ complexes^35^.

**Figure 3:**
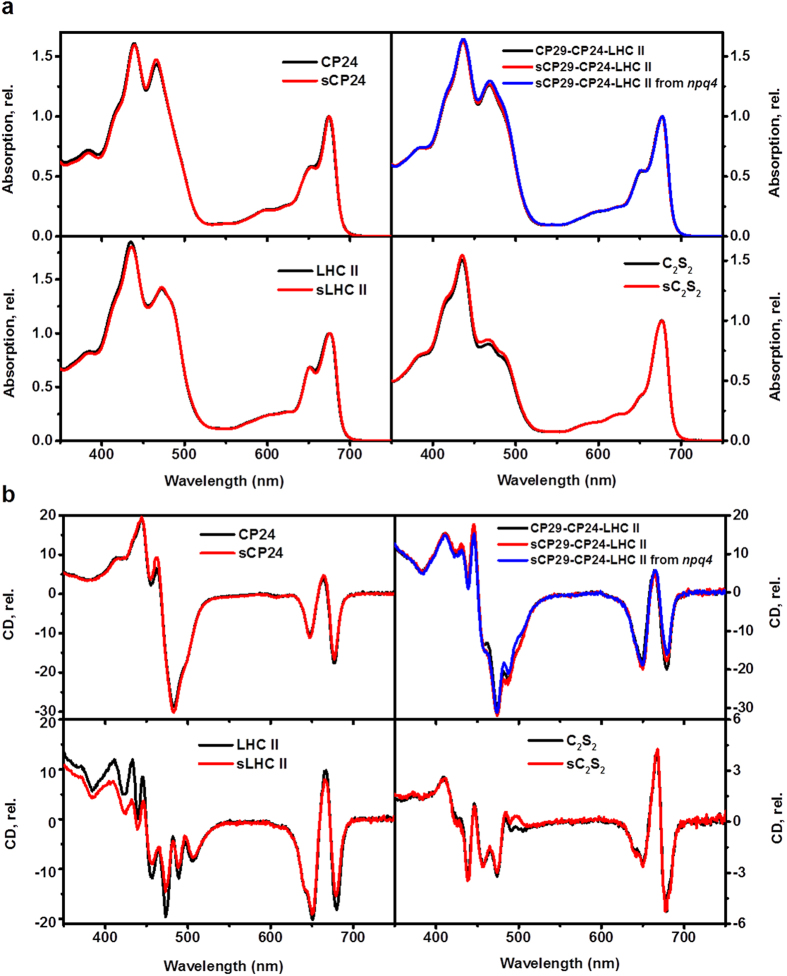
Absorption and Circular Dichroism spectra. (**a**) Absorption and (**b**) Circular Dichroism spectra of pigment-protein complexes and supercomplexes as indicated in the figures. The absorption spectra are normalized to the maximum in the Qy region and the CD spectra are normalized to the absorption.

The purity of the isolated complexes was confirmed by SDS-page (Fig. 2b). The absorption (Fig. 3a) and circular dichroism (CD, Fig. 3b) spectra of the same complexes purified from control and light-stressed plants were virtually identical in the Chl absorption region indicating that the stress does not induce differences in their Chl composition and organization. Small changes were visible in the blue absorption region, in agreement with changes in the carotenoid composition upon light stress.

### How much zeaxanthin is associated with the photosynthetic complexes?

The pigment content of the complexes is reported in Table 1. As expected, the Chl composition of the complexes purified from control and light-stressed plants was nearly identical. It is important to realize that our purification conditions permit retaining practically all carotenoids in all binding sites. As reported in table 1, our purified LHCII have 3.8–3.85 xanthophylls per monomer, indicating that even the loosely bond carotenoids in the V1 site are almost fully retained. The main differences between control and stressed samples concern the carotenoid composition, especially for the presence of Zea in the complexes from stressed plants: 0.44 molecules of Zea per complex were associated with the LHCII trimer and 0.24 with CP24. The amount of Zea was 0.74 in CP29-CP24-LHCII and 2.5 per complex in C_2_S_2_. This difference suggests that some Zea is lost during the purification of the individual components, which implies that this Zea is located at the periphery of the complexes, although we cannot exclude that CP26 has a higher affinity for Zea than the other antennas.

### Where does zeaxanthin bind?

In the *npq*2 *A. thaliana* mutant, which contains Zea constitutively, and in the Lhc reconstituted *in vitro*, Zea binds tightly to the L1 and L2 sites, where it is active in triplet quenching^10^. A clear signature of this can be seen in the triplet-minus-singlet (TmS) spectra, where the contribution of Zea is clearly visible in the 500–530 nm region thanks to its red-shifted spectrum^10^.

To check the presence of Zea in the internal Lhc carotenoid binding sites, we measured the TmS spectra of the complexes purified from control and NPQ conditions. If Zea is located in the central sites we expect a red-shift in triplet signal (around 520 nm) in the spectrum of the stress sample compared to the control. Note that this comparison is possible only if the protein composition of the samples is identical, because the individual complexes have a different TmS spectrum^10^.

**Figure 4:**
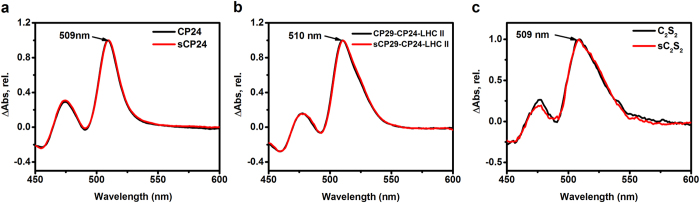
Triplet-minus-singlet (TmS) spectra. The TmS spectra after 100 ns of the samples from control (black) and stress (red) plants are shown normalized to the maximum. (**a**) CP24, (**b**) CP29-CP24-LHCII, (**c**) C_2_S_2_.

The TmS spectra of CP24 with and without Zea as well as those of CP29-CP24-LHCII and C_2_S_2_ were virtually identical (Fig. 4), indicating that Zea does not participate in triplet quenching in the complexes and that its amount in the internal carotenoid binding sites can only be very small, if it is present at all.

### Does zeaxanthin induce quenching in the (super)complexes?

**Figure 5:**
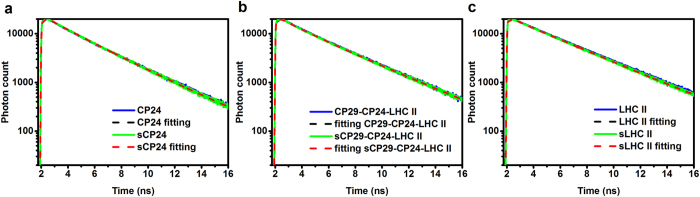
Fluorescence decay kinetics of antenna complexes. The fluorescence decay kinetics of the samples from control and stressed plants are shown. (**a**) CP24; (**b**) CP29-CP24-LHCII and (**c**) LHCII trimers. The weighted residuals of the decay curves fitting are shown in Supplementary Fig. S2.

Recombinant complexes containing Zea and complexes purified from the *npq2* mutant were shown to be in a quenched state^19^,^25^,^26^. To check if this is also the case in native complexes, we have performed time-resolved fluorescence measurements on all purified antennas (CP24, LHCII trimers and CP29-CP24-LHCII), as well as in PSII supercomplexes from light-stressed and control plants. The data for the purified antennas are shown in Fig. 5 and the lifetimes and amplitudes are reported in Table 2. The average lifetimes of all antennas were identical in the presence or absence of Zea. Note that the measurements on the light-stressed samples were done at pH 5.5, a condition that mimics the quenched state *in vivo*. We thus conclude that not only, as we have observed before, the low pH in the absence of aggregation is not able to switch the Lhcs to a quenched conformation^36^, but the switch does not happen even in the presence of Zea, i.e. the Zea associated with the antenna complexes does not induce *per se* quenching in the individual complexes.

**Figure 6:**
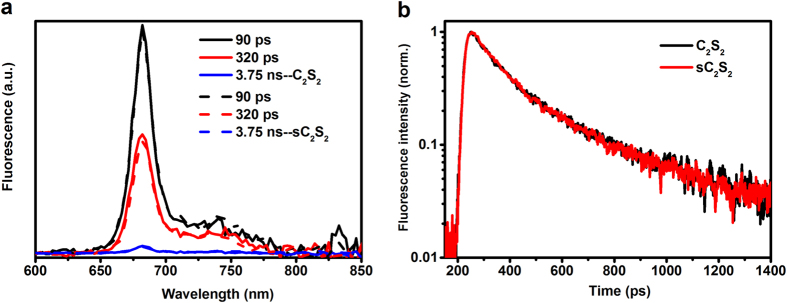
Fluorescence decay kinetics of PSII supercomplexes. Decay associated spectra (DAS) of (**a**) C_2_S_2_ control and stressed and (**b**) Fluorescence decay traces at 680 nm of C_2_S_2_ from control (black) and stress (red) plants.

*In vivo* measurements have led to the suggestion that the zeaxanthin-dependent quenching is active at the level of the C_2_S_2_ complex^33^,^37^. To test if Zea induces quenching in the isolated supercomplexes we have then performed time-resolved fluorescence with a streak camera setup, which allows better resolution of the fast decays, on C_2_S_2_ with and without Zea at pH 5.5. The results presented in Fig. 6 and Table 2 show that the lifetimes of C_2_S_2_ with and without zeaxanthin are identical, leading to the same conclusion as for the individual complexes: No zeaxanthin-induced quenching was observed.

## Discussion

Almost 30 years after the discovery of the correlation between zeaxanthin and NPQ^12^, the real action mechanism of NPQ still remains obscure. It is now accepted that Zea free in the membrane has antioxidant capacity^38^, but the role of this xanthophyll in excited-state energy quenching is less clear. In the current view, Zea is believed to bind to the Lhcs inducing a change in their conformation that leads to a quenched state^15^,^16^,^21^,^24^,^39^. However, the results presented here show that while zeaxanthin-dependent quenching is still active at the level of thylakoid membranes, this is not the case in isolated Lhcs and functional supercomplexes, where the presence of Zea does not have any direct or indirect effect. It is observed that in NPQ conditions Zea does not occupy the internal binding sites L1 and L2 of the Lhcs, which appears as an essential condition for the quenching in individual complexes. Indeed, it should be noted that the experiments supporting binding of Zea to these sites and its relation with the quenching have been performed using complexes purified from the *npq2* mutant, which contains Zea constitutively, or recombinant Lhcs reconstituted in the presence of Zea. In both cases, it was clearly shown that Zea does bind to the L1 and/or L2 sites and its presence stabilizes a quenched conformation. However, the data presented here show that this does not happen, or at best happens with very low frequency, in NPQ conditions. This is not a paradox. The ability of the internal binding site to accommodate Zea is actually not surprising: the internal sites of all Lhcs are promiscuous as they can accommodate most of the xanthophylls present during folding both *in vivo* and *in vitro*^19^,^40^. Moreover, the L1 and L2 sites have a high affinity for Lut, an isomer of Zea^6^,^7^,^40^. The situation under light-stress is different because in this case the complexes are already folded, and Vio would need to be released from the binding sites, de-epoxidized and then Zea would have to be re-inserted in the same sites, which seems hard to achieve^41^. It is possible that upon prolonged high light treatment the newly folded Lhcs also contain Zea in the internal sites, but as the turnover of the antenna is several days^42^, this can only have some influence in long term acclimation.

In the alternative model Zea is considered as an allosteric effector that when bound to the V1 site of LHCII changes the affinity of the protein for protons, shifting the pH-dependence of NPQ to more alkaline values^30^,^43^. It has been argued that once protonated the individual Lhcs assume a quenched conformation without the need of protein-protein interactions or aggregation^44^,^45^ or PsbS^43^. However, our results show that the binding of Zea to Lhcs and supercomplexes does not lead to a quenched conformation, not even at low pH.

If the Zea is not responsible for converting the individual Lhcs to a quenched conformation how then does the zeaxanthin-dependent quenching work? Zeaxanthin-dependent quenching is active in isolated membrane^46^(Fig. 1b), but not in isolated supercomplexes (Fig. 6). The difference in Zea content between supercomplexes and individual antennas, indicates loss of Zea during the purification, suggesting that Zea is accommodated at the periphery of the complexes, not only LHCII but also minor antennas. This association might thus not require only well-defined binding sites (the V1 site is not present in the minors), but Zea can be trapped at the protein-protein interface and might come into close contact with some of the exposed Chls, working in synergy with PsbS and directly or indirectly creating new quenching centers. This hypothesis is supported by the observation that Zea can enhance aggregation as suggested by *in vitro*^21^,^47^,^48^ and *in vivo*^49^ studies, but was also suggested to act directly as a quencher in the membrane^18^. This model is consistent with the fact that zeaxanthin-dependent quenching is active only in the presence of Lhc proteins^50^ but that none of the Lhc KO mutants shows a clear effect on NPQ^35^,^51^,^52^ indicating that none of the antennas is essential for NPQ. It can also explain the presence of several quenching mechanisms observed by *in vivo* measurements, namely Zea radical cation^18^, Chl-Zea interactions^21^ and the Chl-Chl interaction^53^.

## Materials and Methods

*Arabidopsis thaliana* WT-Col 0, CP24ko, *npq2*, *npq4*, CP24-his tag (containing CP24 carrying a His tag at C-terminal, see^8^ for details) plants were grown under 110–130 μmol photons m^−2^ s^−1^ (14 h/day) at 21 °C for 4–6 weeks.

### *In vivo* NPQ measurement

The measurements on thylakoids were performed as previously described^46^. A measuring beam of 5 μmol photons m^−2^ s^−1^, saturating light pulses of 2000 μmol photons m^−2^ s^−1^(duration 500 ms) and actinic light of 531 μmol photons m^−2^ s^−1^ were used. Measurements were done on samples with OD680 nm 0.7–1 (1 cm) in the presence of 30 mM sodium ascorbate and 50 μM methyl viologen. In addition, to discriminate between the zeaxanthin-dependent and ΔpH-dependent quenching, 3 mM DTT was used to inhibit violaxanthin de-epoxidation, while to prevent the formation of pH gradient 3 μM Nigericin were added. All samples were kept in a buffer containing 0.1 M sucrose, 10 mM NaCl, 10 mM KCl, 5 mM MgCl_2_, 10 mM Tricine, 1 mM KH_2_PO_4_, and 2% BAS, pH 8.0^46^ and measured directly after isolation.

### Pigment-protein complexes isolation

Thylakoid/grana membranes preparation were performed as described previously^4^ but D-sorbitol was used instead of NaCl in the buffer during thylakoid preparation. Sucrose gradient were performed as described previously^4^,^8^.

To purify CP24 and the CP29-CP24-LHCII complexes, Band 2 and Band 4 were collected from the sucrose gradients and loaded onto a Ni column^8^. C_2_S_2_ complexes were purified as described previously^4^ but starting from CP24ko grana membranes.

To induce light stress, CP24-His tag, CP24ko, and *npq4* plants were exposed to 1500–1800 μmol photons m^−2^ s^−1^ for 30 minutes. Leaves were then detached from plants in the light and kept in ice/water mixture to inhibit Zea de-epoxidation. Thylakoids/grana membranes were prepared as described above but using 20 mM MES pH 5.5 buffer^39^ in all the purification steps (except the dilution and binding buffers for affinity chromatography). SDS-PAGE was performed as previously described^54^ and stained by Coomassie brilliant blue.

### Absorption and circular dichroism measurement

Room-temperature absorption spectra were measured with a Varian Cary 4000 UV-Vis-spectrophotometer. The Circular-dichroism (CD) spectra were recorded using a Chirascan-Plus spectropolarimeter (Applied Photophysics) at 20 °C. The OD of the samples was 0.8–1/cm at the maximum of the Qy band. All measurements were performed in the same buffers used for the sucrose gradients.

### Pigment analysis

Pigments were extracted from the purified pigment-protein complexes or frozen leaves with 80% acetone. Chl a/b and Chls/carotenoid ratios were calculated by fitting the absorption spectrum of the pigment extract with the spectra of the individual pigments, and the relative amount of carotenoids was determined by HPLC as described previously^55^ but with the modifications. Acetone extracts (~200 μL, Qy OD 0.3–0.8/cm) were loaded on a SephereClone 5u ODS1 column (Phenomenex) under a 1.5 mL/min flow rate. Buffer A was composed of acetonitrile/methanol/0.1 M Tris-HCl pH 7.6 in 72:8:10 ratio. Buffer B was composed of methanol/hexane in 4:1 ratio. The amount of buffer B was linearly increased to 50% in 10 min, held at this value for 0.5 min and then increased linearly to 100% in 4 min. With this method, all photosynthetic pigments can be separated (Supplementary Fig. S1).

### Transient absorption

For the triplet minus singlet measurements, sample concentration was adjusted to an OD of 0.3–0.5/mm at the Qy maximum. The spectra were recorded using a femtosecond transient absorption setup as previously described^56^. Long delays were achieved by using two individual femtosecond amplifiers for pump and probe. Due to the shared source of seed pulses among the two lasers, femtosecond time resolution and arbitrary long pump probe delays were achieved. The excitation wavelength was set at 640 nm with 50–100 nJ per pulse. Measurements were performed in aerobic condition in a shaking cuvette at room temperature. The spectra at 50–400 ns delay which represent the maximum of the carotenoid triplet spectrum were selected.

### Time-resolved fluorescence measurements

Time-Correlated Single Photon Counting (TCSPC) was measured using a FluoTime200 setup (Picoquant). The samples were diluted to an OD of 0.05/cm at the Qy maximum, stirred in a 3.5 ml cuvette with a path length of 1 cm, and kept at 283 K. The fluorescence decay kinetics were detected at 680 nm with a channel time spacing of 8 ps upon 468 nm excitation using a laser diode operating at a repetition rate of 10 MHz. Data analysis was performed by *TRFA DATA processor* using a time windows of 16 ns.

Time-resolved fluorescence decay of PS II supercomplexes were recorded with a Hamamatsu C5680 synchroscan streak camera, combined with a Chromex 250IS spectrograph.

A grating of 50 grooves/mm and blazed wavelength 600 nm was used with the central wavelength set at 720 nm during the measurement. Each image covers a spectral width of 260 nm. Excitation light was vertically polarized, the spot size diameter was typically ~100 μm and the laser repetition rate was tunable from 10 kHz to 300 kHz. Excitation wavelength of 400 nm was used. To avoid photodamage and significant singlet-singlet/triplet annihilations, the sample was magnetically stirred in a cuvette (1 cm × 1 cm × 4 cm) with speed 1500 rpm. ~750 μL solution with OD 3 at 677 nm was used for each sample. To keep the PSII RC open, the laser repetition rate was tuned down to 10 kHz and its laser power was kept as low as 600 nW, in addition, 2 mM ferricyande was added to the sample. The averaged image was corrected for background and shading, and then sliced into traces of ~3 nm width. All measurements were performed at 4 °C.

## Additional Information

**How to cite this article**: Xu, P. *et al.* Molecular insights into Zeaxanthin-dependent quenching in higher plants. *Sci. Rep.* **5**, 13679; doi: 10.1038/srep13679 (2015).

## Supplementary Material

Supplementary Information

## Figures and Tables

**Table 1:** Pigment Composition (the values are normalized to the number of chlorophylls in each complex).

**sample**	**chl a/b**	**chls/cars**	**N**	**V**	**L**	**Z + A**	**Z**	**A**	**β-C**	**Chl b**	**Chl a**	tot chl*	**Tot car**	**(Z + 0.5 × A)/(Z + A + V)**
CP24	1.04	5.22	0.09	0.89	0.89	N.D.	N.D.	N.D.	0.05	4.89	5.11	10	1.92	-
***STDEV***	*0.01*	*0.09*	*0.00*	*0.02*	*0.02*	-	-	-	*0.00*	*0.01*	*0.01*	-	-	-
sCP24	0.99	5.17	0.04	0.75	0.82	0.24	0.20	0.04	0.08	5.02	4.98	10	1.93	0.220
***STDEV***	*0.00*	*0.02*	*0.00*	*0.01*	*0.01*	*0.00*	*0.00*	*0.00*	*0.00*	*0.01*	*0.01*	-	-	-
CP29-CP24-LHC II	1.47	4.05	3.03	2.76	9.32	N.D.	N.D.	N.D.	0.95	26.30	38.70	65	16.06	-
***STDEV***	*0.00*	*0.01*	*0.01*	*0.01*	*0.02*	-	-	-	*0.01*	*0.05*	*0.05*	-	-	-
sCP29-CP24-LHC II	1.43	4.10	3.25	2.15	8.99	0.74	0.56	0.18	0.72	26.77	38.23	65	15.86	0.226
***STDEV***	*0.01*	*0.01*	*0.01*	*0.03*	*0.02*	*0.03*	*0.02*	*0.01*	*0.07*	*0.08*	*0.08*	-	-	-
C_2_S_2_	3.08	4.39	7.45	3.88	24.27	N.D.	N.D.	N.D.	12.21	51.43	158.57	210	47.83	-
***STDEV***	*0.02*	*0.01*	*0.16*	*0.05*	*0.16*	-	-	-	*0.31*	*0.25*	*0.25*	-	-	-
sC_2_S_2_	3.06	4.26	8.84	2.31	22.46	2.50	1.54	0.96	13.27	51.72	158.28	210	49.37	0.421
***STDEV***	*0.02*	*0.13*	*0.28*	*0.07*	*0.62*	*0.22*	*0.16*	*0.06*	*0.35*	*0.22*	*0.22*	-	-	-
LHCII	1.35	3.64	3.01	0.65	7.88	N.D.	N.D.	N.D.	N.D.	17.91	24.09	42	11.54	-
***STDEV***	*0.00*	*0.03*	*0.05*	*0.02*	*0.11*	-	-	-	-	*0.02*	*0.02*	-	-	-
sLHCII	1.33	3.70	2.98	0.28	7.65	0.44	0.33	0.11	N.D.	18.02	23.98	42	11.34	0.534
***STDEV***	*0.01*	*0.05*	*0.06*	*0.03*	*0.11*	*0.00*	*0.00*	*0.01*	-	*0.04*	*0.04*	-	-	-

Legend: N, neoxanthin; V, violaxanthin; L, lutein; Z, zeaxanthin; A, anteraxanthin; β-C, β-Carotene.

*The number of chlorophylls in each complex is based on previous works^4^,^5^,^6^,^7^,^8^. N.D.: non detectable.

**Table 2:** Excited-state lifetimes of the complexes in the presence and absence of zeaxanthin.

**sample**	**τ 1 (ns)**	**A1 (%)**	**τ 2 (ns)**	**A2 (%)**	**τ 3 (ns)**	**A3 (%)**	**Average life time (ns)**
**CP24**	0.304	0.099	1.949	0.270	3.488	0.631	2.759 ± 0.020
**sCP24**	0.329	0.071	1.907	0.246	3.383	0.683	2.802 ± 0.011
**CP29-CP24-LHCII**	0.243	0.163	1.394	0.156	3.654	0.681	2.746 ± 0.006
**sCP29-CP24-LHC II**	0.261	0.130	1.387	0.144	3.635	0.726	2.873 ± 0.020
**LHC II**	0.804	0.062	3.785	0.938	−	−	3.599 ± 0.013
**sLHC II**	0.828	0.072	3.724	0.928	−	−	3.515 ± 0.009
**C2S2**	0.090	0.634	0.320	0.355	3.749	0.011	0.172*
**sC2S2**	0.090	0.660	0.320	0.314	3.749	0.026	0.164*

^*^average of τ 1 and τ 2

## References

[b1] Rochaix J. D. Regulation and dynamics of the light-harvesting system. Annu Rev Plant Biol 65, 287–309 (2014). 24471838 10.1146/annurev-arplant-050213-040226

[b2] Ruban A. V., Johnson M. P. & Duffy C. D. The photoprotective molecular switch in the photosystem II antenna. Biochim Biophys Acta 1817, 167–181 (2012). 21569757 10.1016/j.bbabio.2011.04.007

[b3] Croce R. & van Amerongen H. Natural strategies for photosynthetic light harvesting. Nature chemical biology 10, 492–501 (2014). 24937067 10.1038/nchembio.1555

[b4] Caffarri S., Kouril R., Kereiche S., Boekema E. J. & Croce R. Functional architecture of higher plant photosystem II supercomplexes. Embo J 28, 3052–3063 (2009). 19696744 10.1038/emboj.2009.232 PMC2760109

[b5] Ben-Shem A., Frolow F. & Nelson N. Crystal structure of plant photosystem I. Nature 426, 630–635 (2003). 14668855 10.1038/nature02200

[b6] Liu Z. *et al.* Crystal structure of spinach major light-harvesting complex at 2.72 A resolution. Nature 428, 287–292 (2004). 15029188 10.1038/nature02373

[b7] Pan X. *et al.* Structural insights into energy regulation of light-harvesting complex CP29 from spinach. Nat Struct Mol Biol 18, 309–315 (2011). 21297637 10.1038/nsmb.2008

[b8] Passarini F., Xu P., Caffarri S., Hille J. & Croce R. Towards *in vivo* mutation analysis: knock-out of specific chlorophylls bound to the light-harvesting complexes of Arabidopsis thaliana - the case of CP24 (Lhcb6). Biochim Biophys Acta 1837, 1500–1506 (2014). 24561227 10.1016/j.bbabio.2014.02.012

[b9] Caffarri S., Croce R., Breton J. & Bassi R. The major antenna complex of photosystem II has a xanthophyll binding site not involved in light harvesting. J Biol Chem 276, 35924–35933 (2001). 11454869 10.1074/jbc.M105199200

[b10] Mozzo M., Dall’Osto L., Hienerwadel R., Bassi R. & Croce R. Photoprotection in the antenna complexes of photosystem II: role of individual xanthophylls in chlorophyll triplet quenching. J Biol Chem 283, 6184–6192 (2008). 18079125 10.1074/jbc.M708961200

[b11] Li X. P. *et al.* A pigment-binding protein essential for regulation of photosynthetic light harvesting. Nature 403, 391–395 (2000). 10667783 10.1038/35000131

[b12] Demmig B., Winter K., Kruger A. & Czygan F. C. Photoinhibition and zeaxanthin formation in intact leaves: a possible role of the xanthophyll cycle in the dissipation of excess light energy. Plant Physiol 84, 218–224 (1987). 16665420 10.1104/pp.84.2.218 PMC1056560

[b13] Niyogi K. K., Grossman A. R. & Bjorkman O. Arabidopsis mutants define a central role for the xanthophyll cycle in the regulation of photosynthetic energy conversion. Plant Cell 10, 1121–1134 (1998). 9668132 10.1105/tpc.10.7.1121 PMC144052

[b14] Morosinotto T., Baronio R. & Bassi R. Dynamics of chromophore binding to Lhc proteins *in vivo* and *in vitro* during operation of the xanthophyll cycle. J Biol Chem 277, 36913–36920 (2002). 12114527 10.1074/jbc.M205339200

[b15] Dall’Osto L., Caffarri S. & Bassi R. A mechanism of nonphotochemical energy dissipation, independent from PsbS, revealed by a conformational change in the antenna protein CP26. Plant Cell 17, 1217–1232 (2005). 15749754 10.1105/tpc.104.030601 PMC1087998

[b16] Ruban A. V. & Johnson M. P. Xanthophylls as modulators of membrane protein function. Archives of biochemistry and biophysics 504, 78–85 (2010). 20615387 10.1016/j.abb.2010.06.034

[b17] Horton P., Ruban A. V. & Wentworth M. Allosteric regulation of the light-harvesting system of photosystem II. Philos Trans R Soc Lond B Biol Sci 355, 1361–1370 (2000). 11127991 10.1098/rstb.2000.0698 PMC1692867

[b18] Holt N. E. *et al.* Carotenoid cation formation and the regulation of photosynthetic light harvesting. Science 307, 433–436 (2005). 15662017 10.1126/science.1105833

[b19] Avenson T. J. *et al.* Zeaxanthin radical cation formation in minor light-harvesting complexes of higher plant antenna. J Biol Chem 283, 3550–3558 (2008). 17991753 10.1074/jbc.M705645200

[b20] Ahn T. K. *et al.* Architecture of a charge-transfer state regulating light harvesting in a plant antenna protein. Science 320, 794–797 (2008). 10.1126/science.1154800 18467588

[b21] Bode S. *et al.* On the regulation of photosynthesis by excitonic interactions between carotenoids and chlorophylls. Proc Natl Acad Sci USA 106, 12311–12316 (2009). 19617542 10.1073/pnas.0903536106 PMC2714278

[b22] Wilk L., Grunwald M., Liao P. N., Walla P. J. & Kuhlbrandt W. Direct interaction of the major light-harvesting complex II and PsbS in nonphotochemical quenching. Proc Natl Acad Sci USA 110, 5452–5456 (2013). 23509270 10.1073/pnas.1205561110 PMC3619350

[b23] Jahns P., Wehner A., Paulsen H. & Hobe S. De-epoxidation of violaxanthin after reconstitution into different carotenoid binding sites of light-harvesting complex II. J Biol Chem 276, 22154–22159 (2001). 11301335 10.1074/jbc.M102147200

[b24] Johnson M. P., Perez-Bueno M. L., Zia A., Horton P. & Ruban A. V. The zeaxanthin-independent and zeaxanthin-dependent qE components of nonphotochemical quenching involve common conformational changes within the photosystem II antenna in Arabidopsis. Plant Physiol 149, 1061–1075 (2009). 19011000 10.1104/pp.108.129957 PMC2633848

[b25] Moya I., Silvestri M., Vallon O., Cinque G. & Bassi R. Time-resolved fluorescence analysis of the photosystem II antenna proteins in detergent micelles and liposomes. Biochemistry 40, 12552–12561 (2001). 11601979 10.1021/bi010342x

[b26] Fuciman M. *et al.* Role of xanthophylls in light harvesting in green plants: a spectroscopic investigation of mutant LHCII and Lhcb pigment-protein complexes. J Phys Chem B 116, 3834–3849 (2012). 22372667 10.1021/jp210042z

[b27] Ruban A. V., Lee P. J., Wentworth M., Young A. J. & Horton P. Determination of the stoichiometry and strength of binding of xanthophylls to the photosystem II light harvesting complexes. J Biol Chem 274, 10458–10465 (1999). 10187836 10.1074/jbc.274.15.10458

[b28] Betterle N., Ballottari M., Hienerwadel R., Dall’Osto L. & Bassi R. Dynamics of zeaxanthin binding to the photosystem II monomeric antenna protein Lhcb6 (CP24) and modulation of its photoprotection properties. Archives of biochemistry and biophysics 504, 67–77 (2010). 20494647 10.1016/j.abb.2010.05.016

[b29] Dall’Osto L. *et al.* Zeaxanthin protects plant photosynthesis by modulating chlorophyll triplet yield in specific light-harvesting antenna subunits. J Biol Chem 287, 41820–41834 (2012). 23066020 10.1074/jbc.M112.405498 PMC3516730

[b30] Ruban A. V. & Horton P. The xanthophyll cycle modulates the kinetics of nonphotochemical energy dissipation in isolated light-harvesting complexes, intact chloroplasts, and leaves of spinach. Plant Physiol 119, 531–542 (1999). 9952449 10.1104/pp.119.2.531 PMC32130

[b31] Amarie S. *et al.* Carotenoid radical cations as a probe for the molecular mechanism of nonphotochemical quenching in oxygenic photosynthesis. J Phys Chem B 111, 3481–3487 (2007). 17388511 10.1021/jp066458q

[b32] Jahns P. & Holzwarth A. R. The role of the xanthophyll cycle and of lutein in photoprotection of photosystem II. Biochim Biophys Acta 1817, 182–193 (2012). 21565154 10.1016/j.bbabio.2011.04.012

[b33] Miloslavina Y., de Bianchi S., Dall’Osto L., Bassi R. & Holzwarth A. R. Quenching in Arabidopsis thaliana mutants lacking monomeric antenna proteins of photosystem II. J Biol Chem 286, 36830–36840 (2011). 21844190 10.1074/jbc.M111.273227 PMC3196121

[b34] Betterle N. *et al.* Light-induced dissociation of an antenna hetero-oligomer is needed for non-photochemical quenching induction. J Biol Chem 284, 15255–15266 (2009). 19307183 10.1074/jbc.M808625200 PMC2685706

[b35] Kovacs L. *et al.* Lack of the light-harvesting complex CP24 affects the structure and function of the grana membranes of higher plant chloroplasts. Plant Cell 18, 3106–3120 (2006). 17114352 10.1105/tpc.106.045641 PMC1693946

[b36] Liguori N., Roy L. M., Opacic M., Durand G. & Croce R. Regulation of light harvesting in the green alga Chlamydomonas reinhardtii: the C-terminus of LHCSR is the knob of a dimmer switch. J Am Chem Soc 135, 18339–18342 (2013). 24261574 10.1021/ja4107463

[b37] Holzwarth A. R., Miloslavina Y., Nilkens M. & Jahns P. Identification of two quenching sites active in the regulation of photosynthetic light-harvesting studied by time-resolved fluorescence. Chemical Physics Letters 483, 262–267 (2009).

[b38] Havaux M., Dall’osto L. & Bassi R. Zeaxanthin has enhanced antioxidant capacity with respect to all other xanthophylls in Arabidopsis leaves and functions independent of binding to PSII antennae. Plant Physiol 145, 1506–1520 (2007). 17932304 10.1104/pp.107.108480 PMC2151694

[b39] Jahns P., Latowski D. & Strzalka K. Mechanism and regulation of the violaxanthin cycle: the role of antenna proteins and membrane lipids. Biochim Biophys Acta 1787, 3–14 (2009). 18976630 10.1016/j.bbabio.2008.09.013

[b40] Croce R., Weiss S. & Bassi R. Carotenoid-binding sites of the major light-harvesting complex II of higher plants. J Biol Chem 274, 29613–29623 (1999). 10514429 10.1074/jbc.274.42.29613

[b41] Barros T. & Kuhlbrandt W. Crystallisation, structure and function of plant light-harvesting Complex II. Biochim Biophys Acta 1787, 753–772 (2009). 19327340 10.1016/j.bbabio.2009.03.012

[b42] Dinc E., Ramundo S., Croce R. & Rochaix J. D. Repressible chloroplast gene expression in Chlamydomonas: a new tool for the study of the photosynthetic apparatus. Biochim Biophys Acta 1837, 1548–1552 (2014). 24333785 10.1016/j.bbabio.2013.11.020

[b43] Johnson M. P. & Ruban A. V. Restoration of rapidly reversible photoprotective energy dissipation in the absence of PsbS protein by enhanced DeltapH. J Biol Chem 286, 19973–19981 (2011). 21474447 10.1074/jbc.M111.237255 PMC3103371

[b44] Ilioaia C., Johnson M. P., Horton P. & Ruban A. V. Induction of Efficient Energy Dissipation in the Isolated Light-harvesting Complex of Photosystem II in the Absence of Protein Aggregation. Journal of Biological Chemistry 283, 29505–29512 (2008). 18728016 10.1074/jbc.M802438200 PMC2662029

[b45] Kruger T. P. *et al.* Controlled disorder in plant light-harvesting complex II explains its photoprotective role. Biophys J 102, 2669–2676 (2012). 22713583 10.1016/j.bpj.2012.04.044 PMC3368130

[b46] Gilmore A. M., Shinkarev V. P., Hazlett T. L. & Govindjee G. Quantitative analysis of the effects of intrathylakoid pH and xanthophyll cycle pigments on chlorophyll a fluorescence lifetime distributions and intensity in thylakoids. Biochemistry 37, 13582–13593 (1998). 9753445 10.1021/bi981384x

[b47] Wentworth M., Ruban A. V. & Horton P. Chlorophyll fluorescence quenching in isolated light harvesting complexes induced by zeaxanthin. FEBS Lett 471, 71–74 (2000). 10760515 10.1016/s0014-5793(00)01369-7

[b48] Gruszecki W. I., Grudzinski W., Gospodarek M., Patyra M. & Maksymiec W. Xanthophyll-induced aggregation of LHCII as a switch between light-harvesting and energy dissipation systems. Biochim Biophys Acta 1757, 1504–1511 (2006). 16978579 10.1016/j.bbabio.2006.08.002

[b49] Johnson M. P. *et al.* Photoprotective energy dissipation involves the reorganization of photosystem II light-harvesting complexes in the grana membranes of spinach chloroplasts. Plant Cell 23, 1468–1479 (2011). 21498680 10.1105/tpc.110.081646 PMC3101555

[b50] Dall’Osto L., Cazzaniga S., Havaux M. & Bassi R. Enhanced photoprotection by protein-bound vs free xanthophyll pools: a comparative analysis of chlorophyll b and xanthophyll biosynthesis mutants. Molecular plant 3, 576–593 (2010). 20100799 10.1093/mp/ssp117

[b51] Andersson J. *et al.* Absence of the Lhcb1 and Lhcb2 proteins of the light-harvesting complex of photosystem II - effects on photosynthesis, grana stacking and fitness. Plant J 35, 350–361 (2003). 12887586 10.1046/j.1365-313x.2003.01811.x

[b52] de Bianchi S. *et al.* Arabidopsis mutants deleted in the light-harvesting protein Lhcb4 have a disrupted photosystem II macrostructure and are defective in photoprotection. Plant Cell 23, 2659–2679 (2011). 21803939 10.1105/tpc.111.087320 PMC3226214

[b53] Miloslavina Y. *et al.* Far-red fluorescence: a direct spectroscopic marker for LHCII oligomer formation in non-photochemical quenching. FEBS Lett 582, 3625–3631 (2008). 18834884 10.1016/j.febslet.2008.09.044

[b54] Schagger H. Tricine-SDS-PAGE. Nature protocols 1, 16–22 (2006). 17406207 10.1038/nprot.2006.4

[b55] Croce R., Canino G., Ros F. & Bassi R. Chromophore organization in the higher-plant photosystem II antenna protein CP26. Biochemistry 41, 7334–7343 (2002). 12044165 10.1021/bi0257437

[b56] Kloz M. *et al.* Carotenoid photoprotection in artificial photosynthetic antennas. J Am Chem Soc 133, 7007–7015 (2011). 21491907 10.1021/ja1103553

