# Supplementary material for: Molecular insights into Zeaxanthin-dependent quenching in higher plants
Source: Sci Rep. 2015 Sep 1;5:13679. doi: 10.1038/srep13679 (PMC4555179; doi:10.1038/srep13679)
Supplement: Supplementary Information [file srep13679-s1.pdf]

Molecular insights into Zeaxanthin-dependent quenching in higher plants

Pengqi Xu, Lijin Tian, Miroslav Kloz and Roberta Croce[*]

Biophysics of Photosynthesis, Department of Physics and Astronomy, Faculty of Sciences, VU University Amsterdam and LaserLab Amsterdam. De Boelelaan, 1081, 1081 HV, Amsterdam, The Netherlands
Correspondence should be addressed to R.Croce (r.croce@vu.nl), Tel : +31 20 59 86310

Supplementary Information

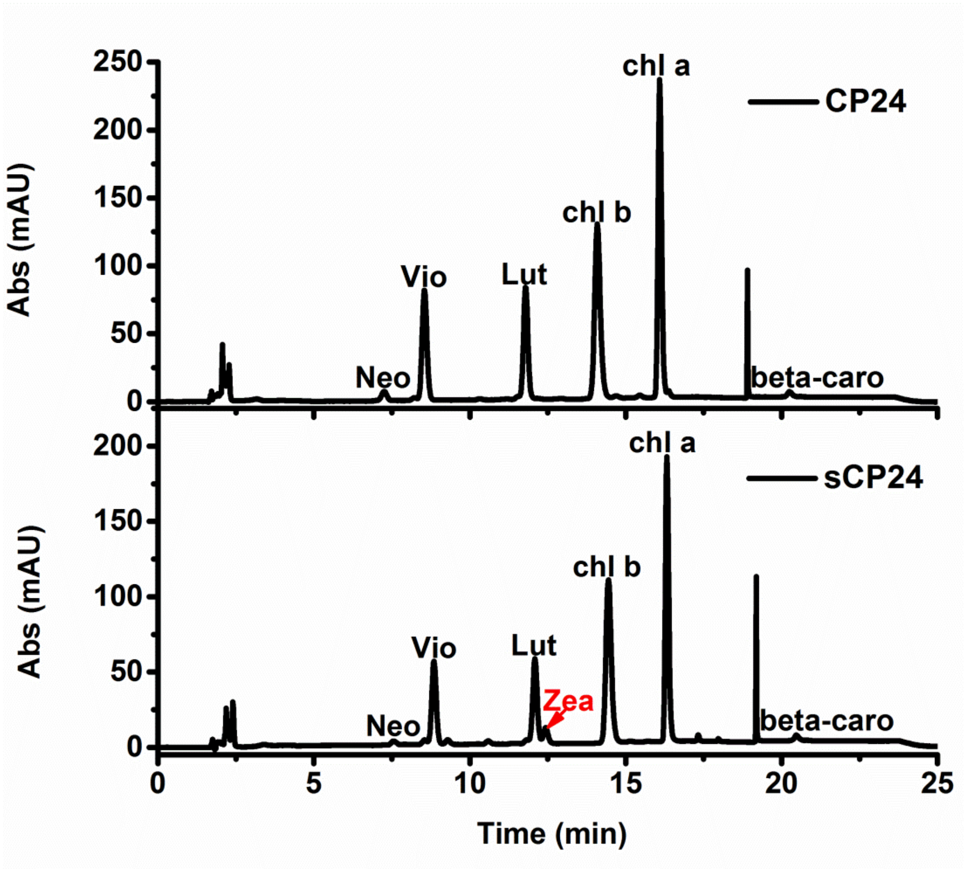

Supplementary Figure S1

Pigments analysis of CP24 and sCP24 (Abs at 440nm).

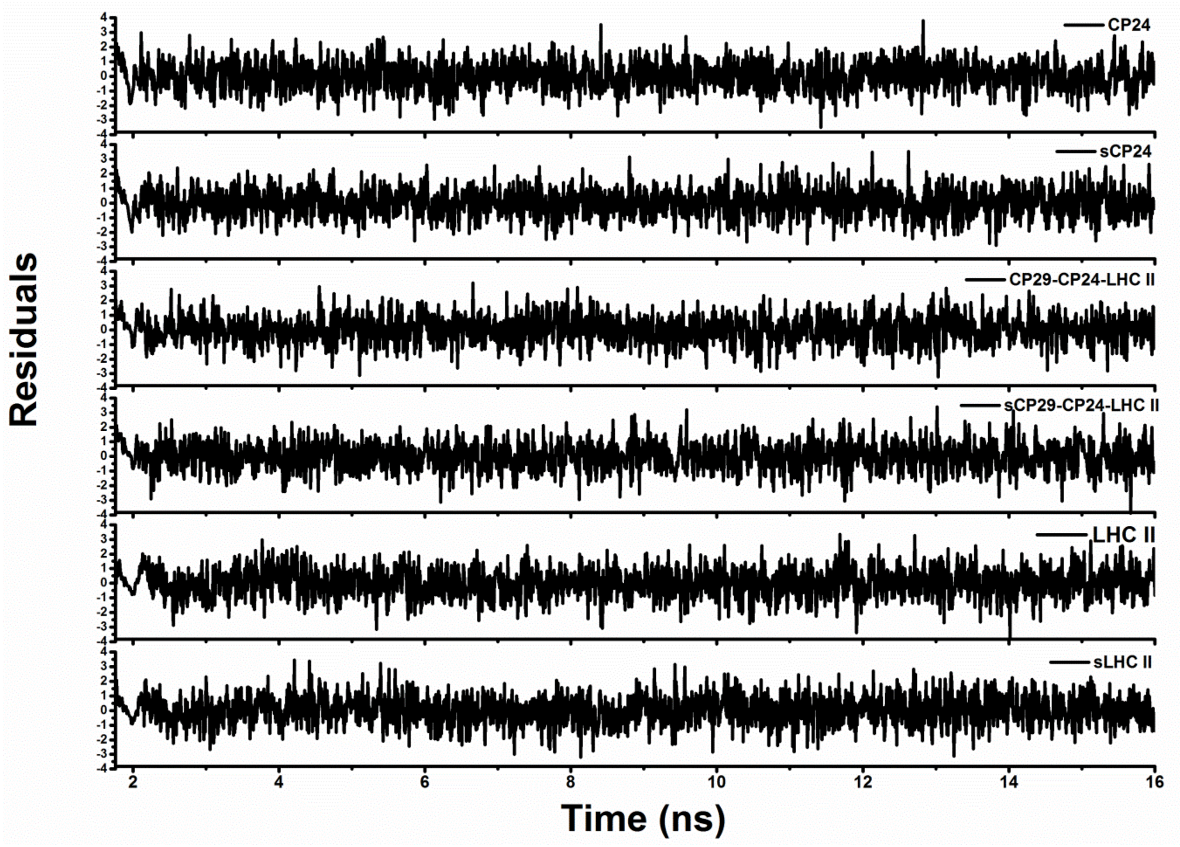

Supplementary Figure S2

Weighted residuals of the fitting of the decay curves in Figures 5.

Supplementary Table S1

Pigments composition of sucrose gradient fractions in control light condition Carotenoids were normalized to 100 total Chls.

sample	Chl a/b	chls/car	N	V	L	β-C
Band 1	5.54	0.44	16.72	44.31	120.55	39.68
STDEV	*0.08*	*0.01*	*1.15*	*2.14*	*5.37*	*1.36*
Band 2	1.80	4.06	4.39	3.41	16.18	0.64
STDEV	*0.00*	*0.00*	*0.26*	*0.10*	*0.15*	*0.02*
Band3	1.40	3.67	7.18	2.14	17.93	
STDEV	*0.00*	*0.01*	*0.03*	*0.02*	*0.03*	
Band 4	1.96	4.12	4.62	3.60	13.77	2.29
STDEV	*0.00*	*0.01*	*0.25*	*0.08*	*0.02*	*0.15*
Band 5	7.85	6.08	1.46	1.25	6.10	7.63
STDEV	*0.06*	*0.03*	*0.05*	*0.05*	*0.06*	*0.24*
Band 6	8.39	4.49	0.45	2.97	6.52	12.32
STDEV	*0.05*	*0.01*	*0.07*	*0.07*	*0.00*	*0.08*
Band 7	7.44	4.61	0.71	2.78	6.55	11.66
STDEV	*0.03*	*0.00*	*0.09*	*0.08*	*0.09*	*0.22*
leaf	3.07	3.73	4.26	3.33	12.62	6.65
STDEV	*0.01*	*0.07*	*0.11*	*0.42*	*0.55*	*0.34*
thy	3.09	3.77	4.05	2.93	12.25	7.32
STDEV	*0.01*	*0.02*	*0.05*	*0.03*	*0.15*	*0.14*

Supplementary Table S2

Pigments composition of sucrose gradient fractions from stress light condition. Carotenoids were normalized to 100 total chls.

sample	chl a/b	chls/ car	N	V	L	Z+A	Z	A	β-C	(Z+0.5×A)/ (Z+A+V)
sBand 1	5.92	0.19	30.21	14.25	393.72	190.20	156.83	33.37	117.70	0.851
STDEV	*0.03*	*0.00*	*0.35*	*0.14*	*2.68*	*1.03*	*0.67*	*0.36*	*0.35*	
sBand 2	1.99	3.38	6.04	1.98	15.60	4.61	3.67	0.94	1.39	0.628
STDEV	*0.01*	*0.02*	*0.21*	*0.07*	*0.15*	*0.11*	*0.12*	*0.05*	*0.14*	
sBand 3	1.41	3.63	7.05	0.53	18.30	1.40	1.07	0.33	0.26	0.640
STDEV	*0.00*	*0.00*	*0.14*	*0.01*	*0.18*	*0.05*	*0.05*	*0.01*	*0.01*	
sBand 4	1.67	3.96	4.26	2.49	15.37	1.69	1.29	0.39	1.44	0.356
STDEV	*0.00*	*0.02*	*0.42*	*0.27*	*0.44*	*0.01*	*0.02*	*0.02*	*0.43*	
sBand 5	5.13	5.49	2.30	1.40	7.97	1.25	0.93	0.31	5.29	0.412
STDEV	*0.03*	*0.02*	*0.10*	*0.68*	*0.34*	*0.05*	*0.02*	*0.03*	*0.24*	
sBand 6 upper	6.42	4.71	0.92	1.75	7.88	1.77	1.27	0.50	8.94	0.432
STDEV	*0.02*	*0.29*	*0.11*	*0.03*	*0.29*	*0.08*	*0.08*	*0.01*	*1.11*	
sBand 6 lower	5.32	5.15	1.74	1.37	8.09	1.44	1.04	0.40	6.80	0.442
STDEV	*0.00*	*0.27*	*0.04*	*0.04*	*0.11*	*0.00*	*0.01*	*0.01*	*0.87*	
sBand 7 upper	4.50	4.93	2.15	1.02	8.27	1.27	0.92	0.35	7.55	0.479
STDEV	*0.01*	*0.07*	*0.03*	*0.03*	*0.04*	*0.02*	*0.01*	*0.01*	*0.30*	
sBand 7 middle	3.53	4.88	2.79	1.03	9.38	1.28	0.92	0.35	6.01	0.478
STDEV	*0.01*	*0.03*	*0.05*	*0.03*	*0.04*	*0.01*	*0.01*	*0.00*	*0.17*	
sBand 7 lower	3.06	4.54	3.17	1.19	10.31	1.36	1.00	0.37	6.01	0.462
STDEV	*0.01*	*0.01*	*0.06*	*0.04*	*0.08*	*0.02*	*0.02*	*0.00*	*0.16*	
sleaf	3.27	3.39	2.64	1.47	13.94	3.67	2.73	0.94	7.77	0.622
STDEV	*0.02*	*0.05*	*0.09*	*0.03*	*0.10*	*0.04*	*0.03*	*0.01*	*0.42*	
sthy	3.30	3.47	4.04	1.37	13.44	2.73	2.15	0.58	7.26	0.596
STDEV	*0.01*	*0.05*	*0.20*	*0.05*	*0.34*	*0.06*	*0.05*	*0.01*	*0.30*	
